# Bacterial Membrane Vesicles as Mediators of Microbe – Microbe and Microbe – Host Community Interactions

**DOI:** 10.3389/fmicb.2020.00432

**Published:** 2020-03-24

**Authors:** Julie C. Caruana, Scott A. Walper

**Affiliations:** ^1^American Society for Engineering Education, Washington, DC, United States; ^2^US Naval Research Laboratory, Center for Biomolecular Science and Engineering, Washington, DC, United States

**Keywords:** outer membrane vesicles, membrane vesicles, community interactions, host-microbe interactions, host-pathogen interactions, cell delivery, antimicrobial

## Abstract

Bacterial membrane vesicles are proteoliposomal nanoparticles produced by both Gram-negative and Gram-positive bacteria. As they originate from the outer surface of the bacteria, their composition and content is generally similar to the parent bacterium’s membrane and cytoplasm. However, there is ample evidence that preferential packaging of proteins, metabolites, and toxins into vesicles does occur. Incorporation into vesicles imparts a number of benefits to the cargo, including protection from degradation by other bacteria, the host organism, or environmental factors, maintenance of a favorable microenvironment for enzymatic activity, and increased potential for long-distance movement. This enables vesicles to serve specialized functions tailored to changing or challenging environments, particularly in regard to microbial community interactions including quorum sensing, biofilm formation, antibiotic resistance, antimicrobial peptide expression and deployment, and nutrient acquisition. Additionally, based on their contents, vesicles play crucial roles in host-microbe interactions as carriers of virulence factors and other modulators of host cell function. Here, we discuss recent advances in our understanding of how vesicles function as signals both within microbial communities and between pathogenic or commensal microbes and their mammalian hosts. We also highlight a few areas that are currently ripe for additional research, including the mechanisms of selective cargo packaging into membrane vesicles and of cargo processing once it enters mammalian host cells, the function of vesicles in transfer of nucleic acids among bacteria, and the possibility of engineering commensal bacteria to deliver cargo of interest to mammalian hosts in a controlled manner.

## Introduction

At both the macro and micro level, life on this planet revolves around complex interactions between individuals, their neighbors, and their environment. At the macro scale researchers study nations, populations, and even communities of humans and animals alike. As we focus in, doctors and scientists examine biological systems, organs, tissues, and individual cells of species to examine how these complex units cooperatively function within a single creature. At an even smaller scale, we can see how these cells and microbes interact both within species and between species to survive in environments that are based on fluctuating models of competition and cooperation. To maintain these balances, cells transmit and receive chemical signals that modulate gene expression and cellular function, transfer biomolecules and metabolites that improve community persistence and viability, and also activate defense strategies that serve to control species competing for the same valuable resources. Many of these biological signaling pathways occur through the cellular synthesis and release of biomolecules into the environment, however, this simplicity is not applicable to all biological systems. Often biomolecules are insoluble, labile, or require specific targeting mechanisms in order to ensure proper transmittance of the signal. In these situations, cells are often able to encapsulate target biomolecules within small proteoliposomes that are loaded and released from the outermost membrane of the parental organism.

**FIGURE 1:**
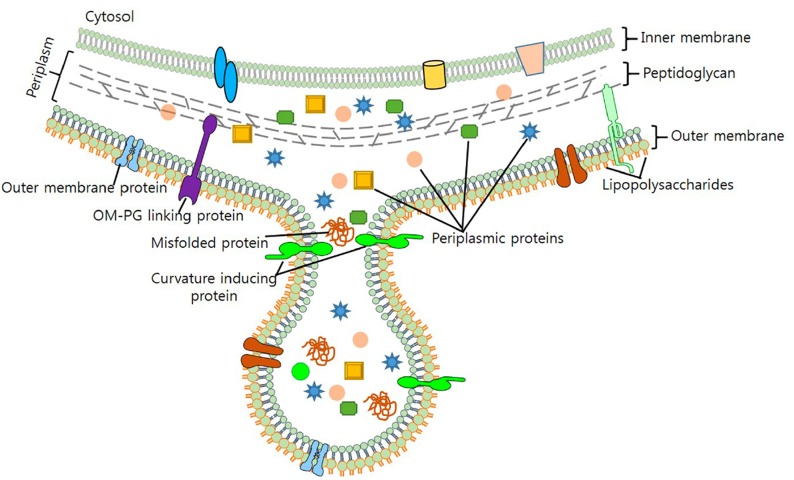
Formation of Gram-negative OMVs. Nascent OMVs form as a constriction of the outermost membrane leading to a blebbing structure that contain both membrane and periplasmic proteins that are encapsulated as cargo. Reproduced with permission from Jan (2017).

Whether stemming from prokaryotes, eukaryotes, or Archaea, most cells studied to date shed portions of their outermost membrane that are loaded with biomolecules. Interestingly, the mechanisms driving vesicle production and release share a number of features across the three domains, underscoring their importance to biological processes (reviewed in Deatherage and Cookson, 2012). Bacterial membrane vesicles, which will be the focus of this review, are referred to as outer membrane vesicles (OMVs) or membrane vesicles (MVs) largely depending upon whether they originate from Gram-negative or Gram-positive bacteria, respectively. Hereafter the term “MVs” will be used to refer to both types of vesicles together or to vesicles specifically from Gram-positive species, while “OMV” will refer specifically to vesicles from Gram-negative bacteria which possess an outer membrane. Bacterial MVs typically range from 25 to 250 nm in diameter and are comprised of and contain within their lumen the proteins, lipids, nucleic acids, and other biomolecules of the parental bacterium (Figure 1; Schwechheimer et al., 2013; Schwechheimer and Kuehn, 2015; Jan, 2017). Once viewed as nothing more than cellular debris or products of membrane rejuvenation, over the past several decades researchers have shown that these biological nanoparticles have a much greater role in cellular function and community interactions. In the subsequent sections, this review will highlight recent discoveries and the understanding they have fostered in the role of bacterial membrane vesicles in microbe-microbe interaction and in interactions between microbial species and the hosts in which they reside (summarized in Table 1).

**TABLE 1:** Membrane vesicle functions in microbe-microbe and microbe-host interactions.

Function	Species	Cargo (if known)	References
**(A) MV functions in interactions within microbial communities**			
OMV biogenesis	*Pseudomonas aeruginosa*	Pseudomonas quinolone signal (PQS)	Mashburn and Whiteley, 2005; Tashiro et al., 2010; Schertzer and Whiteley, 2012
Decoys for titration of harmful substances including antimicrobial peptides, membrane-active antibiotics, phage, chlorhexidine	*Escherichia coli, Moraxella catarrhalis, P. gingivalis*	Unknown factors, LPS (binds chlorhexidine)	Grenier et al., 1995; Manning and Kuehn, 2011; Yamaguchi et al., 2013; Roszkowiak et al., 2019
Quorum sensing	*Pseudomonas aeruginosa, Paracoccus denitrificans, Vibrio harveyi*	Pseudomonas quinolone signal (PQS), C16-HSL, CAI-1	Mashburn and Whiteley, 2005; Toyofuku et al., 2017; Brameyer et al., 2018
Biofilm formation	*Porphyromonas gingivalis, Pseudomonas aeruginosa, Helicobacter pylori*	DNA, other unknown cargo	Singh et al., 1989; Kamaguchi et al., 2003; Allesen-Holm et al., 2006; Yonezawa et al., 2009
Iron acquisition	*Pseudomonas aeruginosa*, *Mycobacterium tuberculosis*	Pseudomonas quinolone signal (PQS), mycobactin (siderophore)	Prados-Rosales et al., 2014; Lin et al., 2017
Polysaccharide metabolism in the human intestine	*Bacteroides spp.*	Polysaccharide utilization loci (PUL) gene products	Elhenawy et al., 2014; Rakoff-Nahoum et al., 2014, 2016; Valguarnera et al., 2018
Cellulose degradation (by horizontal gene transfer)	*Ruminococcus alba*	DNA for cellulolytic genes	Klieve et al., 2005
Antibiotic resistance (enzyme-based)	*Pseudomonas aeruginosa, Moraxella catarrhalis, Staphylococcus aureus, Bacteroides spp., Acinetobacter baumannii, Escherichia coli*	β-lactamases, membrane bound proteases	Ciofu et al., 2000; Schaar et al., 2011; Lee et al., 2013; Stentz et al., 2014; Kulkarni et al., 2015; Liao et al., 2015
Antibiotic and antimicrobial peptide resistance (via horizontal gene transfer)	*Neisseria gonorrhoeae, Escherichia coli, Acinetobacter baumannii*	DNA for β-lactamase genes	Dorward et al., 1989; Yaron et al., 2000; Rumbo et al., 2011; Chatterjee et al., 2017
Protection from oxidative damage	*Helicobacter pylori*	KatA (catalase)	Lekmeechai et al., 2018
Horizontal gene transfer of virulence genes	*Escherichia coli*	DNA for genes encoding intimin and shiga toxin	Kolling and Matthews, 1999; Yaron et al., 2000
Lysis of other microbes (for defense or predation)	*P. aeruginosa, Myxococcus xanthus, Cystobacter velatus*, Strains of *Sorangiineae, Citrobacter, Enterobacter, Escherichia, Klebsiella, Morganella, Proteus, Salmonella*, and *Shigella, Lactobacillus acidophilus* and other lactic acid bacteria	Proteases, hydrolases, secondary metabolites with antimicrobial activity, bacteriocins	Kadurugamuwa and Beveridge, 1996; Li et al., 1996; Mashburn and Whiteley, 2005; Evans et al., 2012; Tashiro et al., 2013; Arthur et al., 2014; Schulz et al., 2018
**(B) MV functions in interaction with mammalian hosts**			
Delivery of toxins and virulence factors to host cells	*Escherichia coli, Shigella dysenteriae, Campylobacter jejuni, Vibrio cholera, Pseudomonas aeruginosa, Porphyromonas gingivalis, Treponema denticola, Helicobacter pylori*	Shiga toxin, hemolysin, cytolethal distending toxin, cholera toxin, alkaline phosphatase, hemolytic phospholipase C, CFTR inhibitory factor, gingipains, dentilisin, vacuolating cytotoxin autotransporter	Rosen et al., 1995; Chi et al., 2003; Dutta et al., 2004; Bomberger et al., 2009; Lindmark et al., 2009; Chatterjee and Chaudhuri, 2011; Choi et al., 2011; Haurat et al., 2011; Bielaszewska et al., 2013; Nakao et al., 2014; Kunsmann et al., 2015; Murase et al., 2015; Zavan et al., 2019
Delivery of nucleic acids to host cells	*Pseudomonas aeruginosa, Aggregatibacter actinomycetemcomitans*, *Porphyromonas gingivalis*, *Treponema denticola*	DNA and RNA	Ghosal et al., 2015; Ho et al., 2015; Sjöström et al., 2015; Koeppen et al., 2016; Bitto et al., 2017; Choi et al., 2017, 2019
Suppression of inflammation related to colitis and allergic responses	*Bacteroides fragilis, Escherichia coli* Nissle 1917, *Bifidobacterium bifidum, Bifidobacterium longum*	Polysaccharide A (PSA), unknown factors	Kalliomäki and Isolauri, 2003; López et al., 2012; Shen et al., 2012; Alvarez et al., 2016; Kim et al., 2016; Fábrega et al., 2017; Ahmadi Badi et al., 2019
Strengthening of intestinal tight junctions and reduced intestinal permeability	*Escherichia coli* Nissle 1917, *Bacteroides vulgaris, Akkermansia spp.*, various *Lactobacillus spp.*	Unknown	Kang et al., 2013; Al-Nedawi et al., 2015; Alvarez et al., 2016; Chelakkot et al., 2018; Maerz et al., 2018; Seo et al., 2018; Yamasaki-Yashiki et al., 2019
Reduced development of obesity and related symptoms in mice fed a high-fat diet	*Akkermansia muciniphila*	Unknown	Ashrafian et al., 2019
Reduction in depressive behavior	*Lactobacillus plantarum*	Unknown	Choi et al., 2019
Prevention or treatment of cancer (cytotoxic effects on hepatic cancer cells)	*Lactobacillus rhamnosus*	Unknown	Behzadi et al., 2017
Protection from pathogen infection (stimulation of host defense genes)	*Lactobacillus plantarum, Lactobacillus sakei*	Unknown	Li et al., 2017; Yamasaki-Yashiki et al., 2019

## Section 1: MVs as Mediators of Microbial Community Interactions

The bulk of research on MV biology has focused on their function in pathogenesis; however, important roles for MVs in microbial community interactions have also been identified and will be described in detail below. Packaging of cargo into MVs allows them to serve specialized functions under changing or challenging environmental conditions including quorum sensing (QS), biofilm formation, nutrient acquisition, antibiotic resistance, and competition with or defense against other microbes. There is evidence that vesiculation is not just a passive process, but rather functions as a controlled mechanism for secretion of cell or envelope components (Schwechheimer et al., 2013; Volgers et al., 2018). For example, the outer membrane protein OmpA is required for maintaining the link between the bacterial outer membrane and peptidoglycan in Gram-negative bacteria, and lower levels of OmpA are correlated with reduced membrane stability and increased vesiculation (Sonntag et al., 1978; Clavel et al., 1998). In *Vibrio cholerae*, the small RNA *vrrA* is upregulated under conditions inducing membrane stress and represses translation of OmpA mRNA. This leads to increased OMV release (inversely correlated with OmpA protein levels) (Song et al., 2008). While the specific mechanisms involved have not yet been fully detailed, packaging of cargo into vesicles appears to occur as both a bulk-flow process as the amount of a specific cargo in the periplasm increases, as well as by preferential packaging method(s). The latter has been shown to be the case for misfolded proteins as a way to selectively eliminate potentially toxic material under stressful conditions (McBroom and Kuehn, 2007). Additionally, selective export of cargo under specific conditions and for specific functions outside the cell has also been demonstrated, and will be discussed in the below sections as it pertains to community interactions among microbes.

### MV Functions in QS and Biofilm Formation

Membrane vesicles play important roles in the dispersal of QS signals, which allow bacteria to communicate with each other and are an important driver of virulence for many pathogens. One of the major QS molecules of *Pseudomonas aeruginosa, Pseudomonas* quinolone signal (PQS), mediates numerous functions including generation of virulence factors, modulation of host immune responses, cytotoxicity against competing microbes, and iron acquisition (Lin et al., 2018). Due to its chemical composition, PQS is highly hydrophobic and thus is not likely to efficiently diffuse through the environment. Instead, it has been shown that about 86% of PQS is packaged into OMVs (Mashburn and Whiteley, 2005). Similarly, the hydrophobic QS molecules C16-HSL of *Paracoccus denitrificans* and CAI-1 of *Vibrio harveyi* are packaged into vesicles, which allows for stabilization, concentration, and dispersal through the environment (Toyofuku et al., 2017; Brameyer et al., 2018). QS mechanisms can also influence OMV production, as PQS is both necessary and sufficient for vesiculogenesis in *P. aeruginosa* and can even induce MV formation in other Gram-negative and even Gram-positive species such as *E. coli, Burkholderia cepacia*, and *B. subtilis* (Mashburn and Whiteley, 2005; Tashiro et al., 2010). The mechanism of OMV biogenesis has been studied in detail and a “bilayer-couple” model has been proposed in which interaction of PQS with the lipid A portion of lipopolysaccharide (LPS) found in the outer leaflet of the bacterial outer membrane causes expansion of the outer leaflet relative to the inner leaflet, resulting in membrane curvature and eventual pinching off of vesicles (Mashburn-Warren et al., 2008; Schertzer and Whiteley, 2012). Additional functions of QS signaling on MV biogenesis and content will be discussed in the later section on host-pathogen interactions.

**FIGURE 2:**
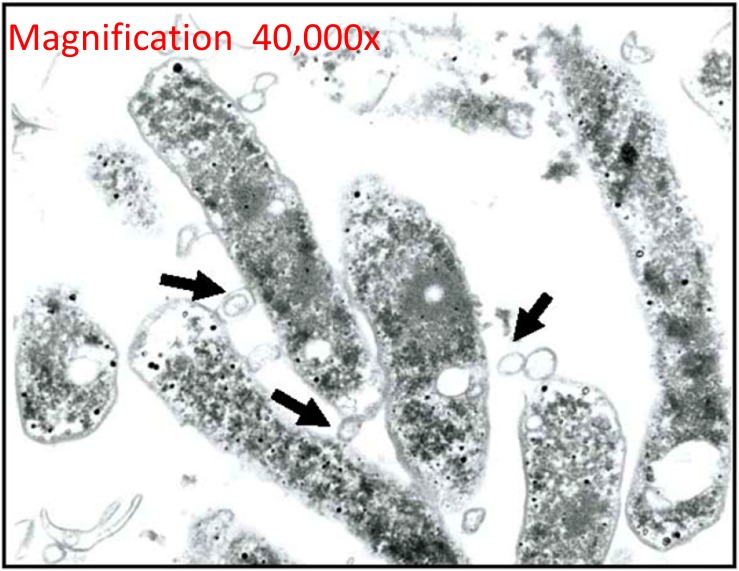
Outer membrane vesicles in *H. pylori* biofilms. TEM micrograph shows the formation and release of OMVs (arrows) of *H. pylori* during the formation of a biofilm in a rich bacterial media. Reproduced with permission (Yonezawa et al., 2009).

Membrane vesicles are an important component of the biofilm matrix for bacterial species, including *P. aeruginosa, Myxococcus xanthus*, and *Helicobacter pylori* (Schooling and Beveridge, 2006; Palsdottir et al., 2009; Yonezawa et al., 2009). As bacterial biofilms are communities that may contain multiple different species, contributions to the biofilm matrix by one species may benefit other species as well and enhance the overall function of the biofilm for cooperation, nutrient acquisition, and enhanced survival (Flemming et al., 2016). In *P. aeruginosa*, quantitative and qualitative differences exist between planktonic- and biofilm-derived OMVs, and they possess proteolytic activity and antibiotic-binding abilities, indicating that they are involved in some of the functions attributed to biofilms (Schooling and Beveridge, 2006). Similar differences have been observed in size and size distribution of planktonic- vs. biofilm-derived MVs of the Gram-positive commensal *Lactobacillus reuteri* and this may indicate differences in function in relation to other members of the microbiome or to the human host (Grande et al., 2017). DNA also functions as a matrix component of biofilms of *P. aeruginosa* and is released specifically in late log-phase cultures in response to QS signals, and this appears to occur at least in part via lysis of DNA-containing OMVs (Allesen-Holm et al., 2006). In *H. pylori*, the strong biofilm-forming ability of strain TK1402 relative to other strains was highly correlated to its production of OMVs, and the addition of the OMV fraction of TK1402 could enhance biofilm formation in another strain (Figure 2; Yonezawa et al., 2009). OMVs from one organism may also facilitate adhesion of another organism in a biofilm; for example, OMVs of the oral pathogen *Porphyromonas gingivalis* could enhance aggregation and adhesion of multiple other oral microorganisms in dental plaque biofilms (Singh et al., 1989; Kamaguchi et al., 2003).

### MV Functions as “Public Goods” for Resource Acquisition

In a microbial community, there is the potential for MVs released by one cell to provide a benefit to other bacteria of the same or of different species. For example, polysaccharide metabolism plays an important role in the establishment and composition of the human intestinal microbiome (Koropatkin et al., 2012). Several species of the genus *Bacteroides* are present, each having different capacities for utilization of the various polysaccharides that reach the human colon intact (Elhenawy et al., 2014). This depends on genes termed polysaccharide utilization loci (PUL), which typically encode surface proteins that can bind, cleave, or import specific polysaccharides and their cleavage products, as well as proteins to further break down those products once inside the cell, and regulatory proteins (Rakoff-Nahoum et al., 2014). Proteomic analysis of OMVs versus the outer membrane (OM) of *B. fragilis* and *B. thetaiotaomicron* identified groups of proteins found exclusively in either the OMVs or the OM, with the OMV-specific proteomes particularly enriched in acidic lipoproteins with hydrolytic or carbohydrate-binding activities that are encoded by PULs (Elhenawy et al., 2014; Valguarnera et al., 2018). These OMVs are able to break down polysaccharides and the resulting products can be consumed by all present bacterial species, even those that did not produce the initial OMVs. This enrichment of PUL-encoded hydrolytic enzymes into OMVs suggests that a mechanism exists to selectively pack certain proteins into the OMVs for function outside the cell, rather than on the cell surface (Elhenawy et al., 2014). Thus, the glycoside hydrolases packaged into OMVs function as “public goods” that benefit the entire bacterial community (Rakoff-Nahoum et al., 2014).

In situations of shared nutrient utilization based on metabolic enzymes exported in MVs, it is not always clear whether the use of these MV-derived nutrients is purely exploitative by recipient cells or if they confer a reciprocal benefit to the MV producers. There is at least one known case of a dedicated cooperative feeding strategy based on OMVs, which occurs between *Bacteroides ovatus* and *Bacteroides vulgatus*, two species that are commonly found together at high densities in the human gut (Rakoff-Nahoum et al., 2014, 2016). *B. ovatus* produces and exports two PUL enzymes for digestion of the polysaccharide inulin inside OMVs, despite the fact that it preferentially imports inulin for intracellular breakdown and extracellular digestion of inulin is not required for optimal *B. ovatus* growth. Additionally, *B. ovatus* exports these enzymes at a cost to itself, as wild type bacteria grow more poorly under conditions inducing inulin hydrolase export than deletion mutants for the two PUL genes. In contrast to *B. ovatus, B. vulgatus* cannot utilize the inulin molecule itself but thrives on inulin breakdown products provided by *B. ovatus* OMVs. *B. ovatus* receives a reciprocal benefit in fitness from *B. vulgatus* through a mechanism that is currently unknown but may be due to *B. vulgatus* production of a growth-promoting factor or detoxification of inhibitory substances. This benefit is specific to the interaction between these two species, as *B. ovatus* does not have increased fitness when it is grown in co-culture with another member of the gut *Bacteroides* community, *B. fragilis.* Thus, OMVs provide a vehicle for a formal cooperative relationship between at least two species in the gut microbial community.

Membrane vesicles can also contribute to nutrient acquisition within a microbial community in other ways. They are vehicles for horizontal gene transfer of genes for metabolic enzymes, as is the case for cellulolytic *Ruminococcus* species in the gut rumen. Klieve et al. (2005) identified linear, double-stranded DNA in MVs of *R. alba*, and hypothesize that chromosomal DNA is specifically processed for packaging into MVs based on its small fragment size, the presence of repetitive DNA sequences that are possibly used for packaging, and its resistance to restriction digestion (perhaps due to modification such as methylation), which they suggest is indicative of DNA intended for export outside the cell. Vesicles from wild type *R. alba* could rescue mutants that are unable to degrade crystalline cellulose and this acquisition of cellulose degradation was heritable, indicating a function of the vesicles in horizontal gene transfer of cellulolytic genes. While cellulolytic bacteria were previously known to produce MVs containing cellulosomes, this report was the first to identify DNA associated with vesicles of *R. alba*, indicating a secondary role of MVs beyond direct cellulose degradation. Further examples of horizontal gene transfer via OMVs are discussed below.

**FIGURE 3:**
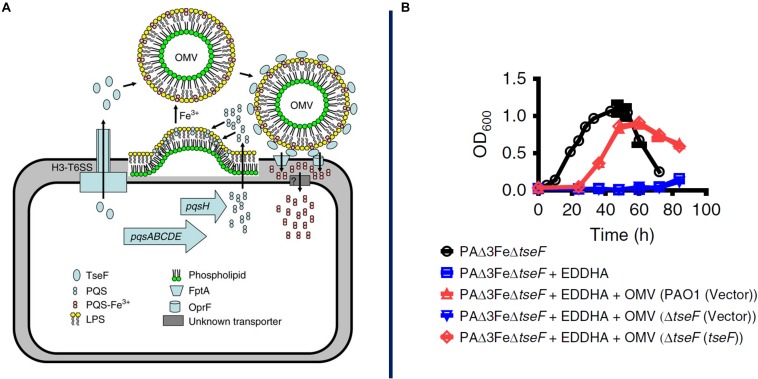
Proposed model for OMV-mediated iron acquisition. Here it is proposed that a soluble protein, TseF, is exported and accumulates on the surface of OMVs containing the QS molecule PQS-Fe^3+^. The TseF protein interacts with cell-surface receptors to facilitate iron uptake into the cell, panel **A**. AS shown in panel **B**, mutants lacking the gene (blue) show a severe growth inhibition which can be recovered through supplementation with OMVs containing TseF protein (red). Reproduced with permission from Lin et al. (2017).

There are multiple examples of MV function in the acquisition of iron, which is essential to bacterial growth but is often limited in the environment due to its poor solubility in water in the presence of oxygen and to active effects by host organisms to sequester it as an immune mechanism to slow pathogen proliferation. For example, *Mycobacterium tuberculosis* increases vesiculation under iron-limited conditions, and these MVs contain high amounts of mycobactin, an iron-chelating protein (siderophore) (Prados-Rosales et al., 2014). Mycobactin is a hydrophobic molecule that accumulates either within or in close proximity to the cell membrane and can then be efficiently incorporated into MVs. Once released into the environment, these MVs can scavenge available iron and then deliver it back to bacterial cells, whether that is the cells that originally produced the MVs or their neighbors. Sequestering mycobactin in MVs may serve a protective function, as in that state it is inaccessible to siderocalin, a siderophore-inhibiting factor released by macrophages during immune responses. MVs purified from cells grown in iron-limited medium can restore growth to mutant cells deficient in siderophore biosynthesis, indicating the potent iron-scavenging ability of these MVs as well as their nature as a community resource (Prados-Rosales et al., 2014). Similarly, the *P. aeruginosa* quorum sensing molecule PQS is primarily exported on the surface of OMVs and can bind Fe^3+^ in the environment (Figure 3; Mashburn and Whiteley, 2005; Lin et al., 2017). OMVs containing the PQS-Fe^3+^ complex are then recaptured by TseF, a type 6 secretion system (T6SS) – exported protein that binds PQS and bridges interaction with siderophore receptors on the cell surface (Lin et al., 2017). As with mycobactin, PQS is a hydrophobic molecule and OMVs provide a vehicle for protection and dispersal through the environment as well as a mechanism to traffic an essential element through a microbial population.

As a counterpoint to the evidence for MV function as public goods that benefit an entire bacterial community, there is also evidence that MV interaction with bacterial cells can be selective. OMVs derived from the enterobacterium *Buttiauxella agrestis* specifically interact with cells of the same or other *Buttiauxella* species, as opposed to with *E. coli* (Tashiro et al., 2017). This is hypothesized to be due to the particular physiochemical properties of cells and OMVs of these species, as *Buttiauxella* spp. have significantly lower zeta potential as compared to many other gram-negative bacteria (producing less electrostatic repulsion between cells and OMVs), as well as to an as-yet-unidentified cell surface protein that may facilitate a specific OMV-cell interaction (Tashiro et al., 2017). Further examination of this specificity in MV-cell interaction could lead to strategies for directed MV-based delivery of cargo to target bacterial cells for biotechnological purposes.

### MV Functions in Microbial Defense

**FIGURE 4:**
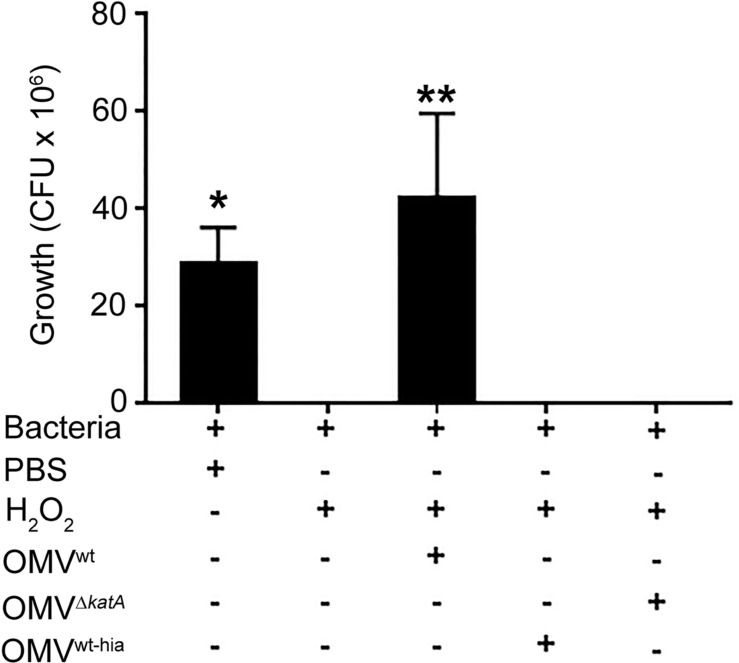
Outer membrane vesicle-mediated protection from environmental stresses. *H. pylori* and other bacterial species often load enzymes capable of degrading reactive oxygen species such as hydrogen peroxide and other environmental compounds. Lekmeechai et al. (2018) showed that OMVs of wild-type *H. pylori* (which contain the catalase *KatA*) could protect *H. pylori* mutants lacking *KatA* from the bactericidal effects of H_2_O_2_, while OMVs from *katA* mutants or heat-inactivated OMVs could not. Data is shown as mean ± SD count of colony forming units (CFU); *n* = 3, *p < 0.05, **p < 0.01. Reproduced with permission from Lekmeechai et al. (2018).

Membrane vesicles can also provide protective function to a microbial community against harmful substances such as reactive oxygen species, antibiotics, antimicrobial peptides, and phage. In many cases, this again occurs through MVs functioning as “public goods” as described in the above section. For example, in *H. pylori*, OMVs are deployed as a protective mechanism against reactive oxygen species released from host immune cells (Figure 4). OMVs of multiple strains are selectively enriched in the catalase KatA as compared to the bacterial outer membrane, and these OMVs display greater activity for H_2_O_2_ hydrolysis than whole cell lysates, which serves to protect the surrounding bacteria from oxidative damage (Lekmeechai et al., 2018). Similarly, strains of *Acinetobacter baumannii* that are resistant to the antibiotic carbapenem selectively release the carbapenem-hydrolyzing enzyme OXA-58 in OMVs, which serves to shelter both the producing strains as well as coexisting carbapenem-susceptible bacteria (Liao et al., 2015). Release of β-lactamases in MVs has been demonstrated for several other species and these MVs have protective effects on other bacteria that may coinhabit their respective communities, including *P. aeruginosa, Moraxella catarrhalis* (protects *Streptococcus pneumoniae* and *Haemophilus influenzae*), *Staphylococcus aureus* (protects *E. coli, Salmonella enterica*, and other *Staphylococcus* strains), and several *Bacteroides* species (protect *Salmonella typhimurium* and the commensal *Bifidobacterium breve*) (Ciofu et al., 2000; Schaar et al., 2011; Lee et al., 2013; Stentz et al., 2014). This effect is not just limited to β-lactamases, as *E. coli* OMVs provide resistance to both the producer *E. coli* strain and to *P. aeruginosa* and *Acinetobacter radioresistens* to the antimicrobial peptide melittin, and this is hypothesized to be due to the presence of membrane-bound proteases on the surface of the OMVs that degrade melittin in the environment (Kulkarni et al., 2015).

In addition to carrying enzymes for benefit of the microbial community, MVs can also serve as vehicles for horizontal gene transfer. *A. baumannii* has been shown to package DNA encoding multiple β-lactamase genes into OMVs, which allows both intra- and interspecies transfer (to *E. coli*) of resistance (Rumbo et al., 2011; Chatterjee et al., 2017). Similarly, transfer of a penicillin resistance gene has been demonstrated in *Neisseria gonorrhoeae* OMVs (Dorward et al., 1989). Yaron et al. (2000) studied the transfer of genetic material in *E. coli* OMVs in detail, and found that virulent *E. coli* O157:H7 packages DNA in the form of both linear chromosomal DNA and circular plasmids. This DNA could then be transferred via OMVs to a non-virulent *E. coli* strain, resulting in antibiotic resistance and increased virulence (Yaron et al., 2000). Transfer of other genes besides those for antibiotic resistance has also been demonstrated, including virulence genes encoding intimin (required for attachment of bacteria to host epithelial cells) and shiga toxin in *E. coli* OMVs (Kolling and Matthews, 1999). There may be a mechanism for selective packaging of particular DNA sequences into vesicles, as DNA found in *P. aeruginosa* OMVs is enriched in genes involved in particular biological functions including antibiotic resistance, survival under stress conditions, metabolism, and membrane synthesis (Bitto et al., 2017).

In the above examples, MVs provide a protective effect based on cargo that is specifically packaged inside them. In other cases, protection conferred by MVs is due to their ability to act as “decoys” for harmful substances, especially membrane-active antibiotics. Multiple strains of *E. coli* (a clinical enterotoxigenic isolate or the laboratory strain K12) increase vesiculation when exposed to the antimicrobial peptides (AMP) polymyxin B and colistin, which exert their effects by forming pores in the bacterial outer membrane. The addition of purified OMVs of either of these strains to cultures at the same time as AMP treatment results in increased bacterial survival, due to their similarity to the native bacterial outer membrane and ability to interact with the AMPs and titrate them out of the environment (Manning and Kuehn, 2011). Similar results were observed by Roszkowiak et al. (2019), who demonstrated that OMVs produced by the bacterial pathogen *M. catarrhalis* could provide passive protection against polymyxin B to *H. influenza, P. aeruginosa*, and *A. baumannii*, as well as the fungal pathogen *Candida albicans*. As polymyxin B can also be a potent antifungal treatment when it is administered with fluconazole, this finding may have important implications when it comes to treating fungal infections at sites where bacterial biofilms may also be involved. OMVs also provide resistance to T4 bacteriophage, again by serving as decoys and binding to the phage before they can reach and infect the bacterial cells (Manning and Kuehn, 2011). Similarly, biofilms of *P. gingivalis* are known to persist after treatment with the antiseptic chlorhexidine, and this is likely due to the fact that LPS found in *P. gingivalis* OMVs can bind to chlorhexidine (Grenier et al., 1995; Yamaguchi et al., 2013). As this protective effect would extend to all bacterial species present in a biofilm, this contributes to the role of *P. gingivalis* as one of the keystone species of chronic periodontitis.

### MV Functions in Antagonistic Interactions Between Microbes

While the above examples describe situations where MVs provide benefits to other bacteria in the community, the opposite case also exists in which they can be used as weapons against other microbes. This has been well studied in the case of *P. aeruginosa*, which produces OMVs containing multiple virulence factors for killing host cells or other bacteria including proteases, hemolysin, phospholipase C, alkaline phosphatase, antibacterial quinolones, and murein hydrolases (Li et al., 1996; Mashburn and Whiteley, 2005; Tashiro et al., 2013). MVs from strains of *Citrobacter, Enterobacter, Escherichia, Klebsiella, Morganella, Proteus, Pseudomonas, Salmonella*, and *Shigella* have also been shown to lyse various Gram-positive and Gram-negative bacteria (Kadurugamuwa and Beveridge, 1996; Li et al., 1998). For Gram-positive bacteria, OMVs attach to the cell wall surface and release peptidoglycan hydrolases, which digest the peptidoglycan cell wall and lead to lysis (Kadurugamuwa and Beveridge, 1996; Kadurugamuwa et al., 1998). Against other Gram-negative bacteria, OMVs produced by Gram-negatives fuse with the outer membrane and release their contents into the host’s periplasm to cause lysis (Li et al., 1998).

In the predatory bacterium *Myxococcus xanthus*, OMVs may play a role in lysing prey cells. *M. xanthus* produces abundant OMVs that contain proteases, hydrolases, and secondary metabolites with antimicrobial activity (Evans et al., 2012; Berleman et al., 2014). Of the identified OMV cargo, alkaline phosphatase is almost exclusively associated with OMVs, suggesting that it is actively packaged, while other OMV cargo appears to be packaged passively as it is found at similar levels in OMVs and parent cells (Evans et al., 2012). These OMVs are thought to fuse with the outer membrane of prey organisms such as *E. coli*, as *M. xanthus* also secretes glyceraldehyde-3-phosphate dehydrogenase (GAPDH) which stimulates membrane fusion. Predation by *M. xanthus* occurs at the community level, as a population of cells secretes antimicrobial substances (free or in OMVs) into the environment, and the resulting lysis products of prey cells can be taken up by the population of predator cells (Marshall and Whitworth, 2019).

Two other species of myxobacteria, *Cystobacter velatus* Cbv34 and *Sorangiineae* species strain SBSr073, have also been shown to produce OMVs that contain antimicrobial factors that inhibit the growth of *E. coli* (Schulz et al., 2018). As myxobacteria are non-pathogenic soil bacteria, their OMVs are biocompatible and showed low endotoxin activity and low acute inflammatory properties when added to human intestinal epithelial cells. OMVs of Cbv34 inhibited *E. coli* growth as well as the established antibiotic gentamicin, making them a promising avenue for further study as a potential alternative to traditional antibiotics. Two bacteriocins produced by lactic acid bacteria, nisin and pediocin PA-1, are used in the food industry to inhibit bacterial contamination and are of interest for clinical antibacterial use; however, their transfer to clinical use has been impeded by the lack of a reliable delivery system (Arthur et al., 2014). As most Lactobacillus species are non-pathogenic, generally regarded as safe, and naturally found in the human microbiome; the possibility exists that MVs of these bacteria could be used for bacteriocin delivery for therapeutic purposes as an alternative to traditional antibiotics once they are further characterized.

## Section 2: Interaction of MVs With Mammalian Host Cells

Due to the importance of MVs in pathogenesis, interaction, and communication between microorganisms and their hosts (mammalian and otherwise), a significant amount of work has been dedicated to understanding the function of MVs in these contexts. However, large gaps remain in our understanding of the mechanisms of cargo packaging into MVs, their uptake into host cells, and the ultimate function and fate of their contents after uptake. In this section, we will discuss our current understanding of how MVs function in both pathogenic and beneficial contexts, with emphasis on the mechanisms of cargo packaging and delivery into host cells. This topic has also been recently reviewed by other researchers and their work may be referred to for additional information (O’Donoghue and Krachler, 2016; Lynch and Alegado, 2017; Tsatsaronis et al., 2018; Cecil et al., 2019).

### Membrane Vesicle Function in Pathogenesis

The capacity for MVs to carry and deliver virulence factors into host cells has made them the subject of extensive research in the context of a number of diseases. As they possess mucosal surfaces which serve as battlegrounds of interaction between pathogens and mammalian hosts, the lungs, oral cavity, and digestive tract and the pathogens associated with these tissues have seen the most study to date. Consequently, much of our understanding of the mechanisms of MV action comes from studies of pathogenic organisms including *P. aeruginosa, P. gingivalis, Vibrio cholerae, H. pylori*, and *E. coli*.

It has long been known that MVs of various pathogens serve as delivery vehicles for virulence factors and toxins to host cells. For example, OMVs of pathogens of the gastrointestinal tract have been shown to contain toxins that lead to cellular distension and lysis in the human intestinal epithelium, such as Shiga toxin and hemolysin (*E. coli, Shigella dysenteriae*), cytolethal distending toxin (*E. coli*, *Campylobacter jejuni*), and cholera toxin (*V. cholerae*) (Dutta et al., 2004; Lindmark et al., 2009; Chatterjee and Chaudhuri, 2011; Bielaszewska et al., 2013; Kunsmann et al., 2015). *P. aeruginosa* packages virulence factors including alkaline phosphatase, β-lactamase, hemolytic phospholipase C and CFTR inhibitory factor into its OMVs, which have varying functions including biofilm formation, degradation of host antimicrobial peptides, cytotoxicity, and inhibition of chloride secretion in the airways (Bomberger et al., 2009; Choi et al., 2011). Oral pathogens such as *P. gingivalis* and *Treponema denticola* secrete virulence factors such as gingipains and dentilisin in their OMVs, which can induce detachment or disrupt tight junctions of oral squamous epithelial cells, facilitating bacterial penetration (Rosen et al., 1995; Chi et al., 2003; Nakao et al., 2014). This list is by no means exhaustive, but gives an idea of the variety of pathogens that employ vesicles as delivery vehicles and the breadth of functions carried out by these virulence factors.

A subset of vesicle cargo contributing to pathogenesis that deserves special mention is that of nucleic acids packaged into MVs. Study of the presence and function of DNA and especially RNA in vesicles has accelerated in recent years and nucleic acids have been discovered in the vesicles of Gram-negative and Gram-positive bacteria, fungi, protists, and archaea (reviewed extensively in Tsatsaronis et al., 2018). As is the case for protein cargo, vesicles represent an ideal delivery mechanism for microbial RNAs as they can protect them from degradation by extracellular RNAses, serve as a vehicle for transport across distances within a host, and deliver them into host cells. Many of the identified nucleic acids packaged in MVs are small RNAs that derive from intergenic regions of the bacterial genome or from non-coding RNAs such as tRNA (Ghosal et al., 2015; Ho et al., 2015; Sjöström et al., 2015; Choi et al., 2017). It has been demonstrated that some of these RNAs can function similarly to miRNAs and direct silencing of target host genes. For example, *P. aeruginosa* packages an sRNA derived from tRNA coding for methionine (tRNA-Met) into OMVs, which then deliver it into lung cells. Once delivered, it modifies the host immune response by suppressing expression of MAP kinases and thus reducing OMV-induced IL-8 secretion in human bronchial epithelial cells and in the lungs of mice (Koeppen et al., 2016). This was the first example of *trans*-kingdom delivery of bacterial regulatory RNA via vesicles, and since then similar discoveries have been made of reduced cytokine production caused by regulatory sRNAs in OMVs of the periodontal pathogens *Aggregatibacter actinomycetemcomitans*, *P. gingivalis*, and *Treponema denticola* (Choi et al., 2017). Very recently, RNA carried in *A. actinomycetemcomitans* OMVs was additionally shown to activate the pro-inflammatory cytokine TNF-α via the TLR-8 and NF-κB signaling pathways in human macrophages (Choi et al., 2019). As periodontal pathogens are thought to contribute to neuroinflammatory diseases including Alzheimer’s disease and OMVs of these species can cross the blood-brain barrier, the possibility exists that pathogenic sRNAs may be behind some aspects of disease development (Choi et al., 2019). These advances in our understanding of the function of regulatory RNAs in vesicles are very recent, but highlight the need for more research in this area and further illustrate the breadth of functions carried out by MV cargo.

### Cargo Packaging in MVs

As in the context of communication between different microbial species, there is ample evidence of differential packaging of cargo into MVs depending on the bacterial growth stage, environment within the host, and status of the microbial community. The following section will highlight various examples of differential OMV production and content to illustrate the variety of behaviors that have been identified. Importantly, evidence in this area has been gathered for various bacterial species on an individual basis, and whether or not any of these behaviors might be widespread and function in additional species is unknown. Additionally, the specific mechanisms underlying each of these cases are still unclear for the most part. Thus, the control processes behind how MV number and content is regulated represent a major area in which further research is needed.

Analysis of *H. pylori* OMVs revealed that the size and protein content is variable depending on the bacterial growth stage (Zavan et al., 2019). As growth progresses, OMVs increase in number, become less heterogeneous in size, and mediate a stronger pro-inflammatory response in human epithelial cells as measured by interleukin-8 (IL-8) production. As described by the authors, OMVs isolated from three different time points contained proteins that were not found in the parent bacteria, indicating that cargo proteins were selectively packaged into OMVs. Proteins involved in metabolic pathways, metabolism in diverse environments, and amino acid transport were more abundant in later-stage OMVs, while OMVs from earlier time points were more enriched in virulence factors including vacuolating cytotoxin autotransporter (VacA), urease, and cag pathogenicity island proteins (Zavan et al., 2019). This variation in OMV size and composition highlights the dynamic nature of OMVs and their potential flexibility as mediators of infection or of communication between organisms.

Outer membrane vesicle content can also be altered in response to environmental conditions. For example, *C. jejuni* is considered a commensal organism in avian hosts, but is pathogenic and causes bacterial gastroenteritis in humans. It was previously shown by proteomics analyses of cultures grown at 42°C (avian body temperature) vs. 37°C (human body temperature) that growth at 37°C results in increased expression of proteins involved in colonization (Zhang et al., 2009). Recently, proteomic analysis of the OMVs themselves identified numerous proteins with differential abundance between the two temperatures, with significantly higher amounts of proteins associated with virulence found in the OMVs from the 37°C culture (Taheri et al., 2019). Comparison between the OMV proteome and the previously published bacterial proteome indicates that the OMV proteome is significantly different from that of the parent cells, again suggesting that a mechanism exists for specifically loading proteins into the OMVs (Zhang et al., 2009; Taheri et al., 2019). Additionally, OMVs from 37°C cultures could induce greater inflammation in mouse bone marrow-derived macrophages as measured by IL-1β activation, supporting a role for temperature in influencing the cargo of *C. jejuni* OMVs and their role in infection (Taheri et al., 2019).

There is also evidence that QS plays a role in the regulation of the production and content of MVs, and this can assist in evasion of host immune responses. One avenue for innate immune response activation is through the detection of conserved pathogenic motifs called pathogen-associated molecular patterns (PAMPs) such as flagellin, LPS, or peptidoglycan by pattern recognition receptors (PRRs) either on the surface of cells such as the Toll-like Receptors (TLRs) or within cells such as the NOD-like receptors NOD1 and NOD2. NOD1 is highly expressed in intestinal epithelial cells, and specifically recognizes a particular moiety found in peptidoglycan of Gram-negative bacteria (Chamaillard et al., 2003). Peptidoglycan has been found in the OMVs of several pathogens including *H. pylori, P. aeruginosa*, and *Neisseria gonorrhoeae* and activates NOD1-dependent immune responses after these OMVs enter intestinal epithelial cells via lipid rafts (Kaparakis et al., 2010). In contrast, certain *V. cholerae* strains of the NOVC serogroups are able to attenuate the inflammatory potential of their OMVs by reducing peptidoglycan packaging into OMVs in a QS-dependent manner (Bielig et al., 2011a, b). At low cell densities, the virulence gene repressor HapR is inactive, which allows expression of virulence factors such as cholera toxin and genes involved in biofilm formation, and also prevents accumulation of peptidoglycan in OMVs. At high cell densities, HapR is stable and virulence genes are repressed, and peptidoglycan is found in OMVs. This repression of virulence factors at high cell densities has been proposed to help cells detach and find a new site of infection or a new host, as would be expected to occur at later stages of infection (Liu et al., 2008). NOVC serogroups of *V. cholerae* lack a number of important virulence factors but are still able to cause sporadic outbreaks, and this may be due in part to their ability to evade NOD1-dependent immune activation (Bielig et al., 2011b).

Aside from the QS effects on MV production described above, there is also evidence for other virulence factors driving OMV biogenesis and cargo sorting. The first virulence factor shown to be directly involved in the production of OMVs was HlyF, a plasmid-encoded protein carried by certain strains of pathogenic *E. coli* (Murase et al., 2015). Strains engineered to express HlyF produce more OMVs than the control strain in which the *hlyF* gene is disrupted, and these OMVs contain increased amounts of the virulence factors ClyA and CDT. Culture supernatants from HlyF-producing strains can induce autophagy in human cell lines, and expression of HlyF contributes to significantly increased virulence in a chicken model (Murase et al., 2015). The exact mechanism by which HlyF drives OMV biogenesis has not yet been determined, but its function was shown to be dependent on a putative catalytic domain indicative of proteins in the short-chain dehydrogenase/reductase (SDR) family (Kavanagh et al., 2008; Murase et al., 2015). SDRs constitute a large family of enzymes involved in lipid, amino acid, carbohydrate, and cofactor metabolism as well as redox sensor mechanisms, thus further work will be required to determine the specific function of HlyF in OMV production (Kavanagh et al., 2008).

Preferential packaging of outer membrane proteins into OMVs has also been shown for the human oral pathogen *P. gingivalis.* Virulence factors such as gingipains are enriched in OMVs, while other abundant outer membrane proteins that do not contribute to infection are excluded, along with periplasmic and cytoplasmic proteins (Haurat et al., 2011). Again, the exact mechanism by which this selective sorting of proteins occurs is not yet known, though it was shown to require the LPS variant containing the negatively charged O-antigen (A-LPS). Haurat et al. (2011) propose a mechanism by which the outer membrane is organized into patches of different LPS molecules sorted by polysaccharide composition or length. Outer membrane proteins are selectively recruited or excluded from those patches based on LPS content, and OMVs could then be produced from those regions that contain selectively packaged proteins (Haurat et al., 2011).

A fascinating example of controlled membrane vesicle production occurs in *M. tuberculosis.* It has coevolved with human hosts to elicit a balanced host immune response, which is enough to restrict pathogen growth but rarely, if ever, completely eliminates the bacteria. This allows it to persist in a latent state in up to one third of the human population, in which it sometimes activates a stronger adaptive immune response that contributes to tissue damage and subsequent transmission to other host individuals (Ernst, 2012; Rath et al., 2013). MVs of *M. tuberculosis* are abundant in factors that bind TLR2 receptors on host macrophages and trigger an immune response; thus, control of MV production is critical to maintaining a low profile. Rath et al. (2013) identified VirR, a membrane-associated protein that restricts vesiculogenesis via an as-yet undetermined mechanism. As MVs from bacteria lacking VirR are hyperinflammatory, this raises the possibility for development of improved vaccines from VirR mutant strains, either in the form of whole cells carrying virulence-attenuating mutations, or of an MV-based vaccine that would be a potent immunogen in non-replicative form (Rath et al., 2013).

### Entry of MVs Into Host Cells

**FIGURE 5:**
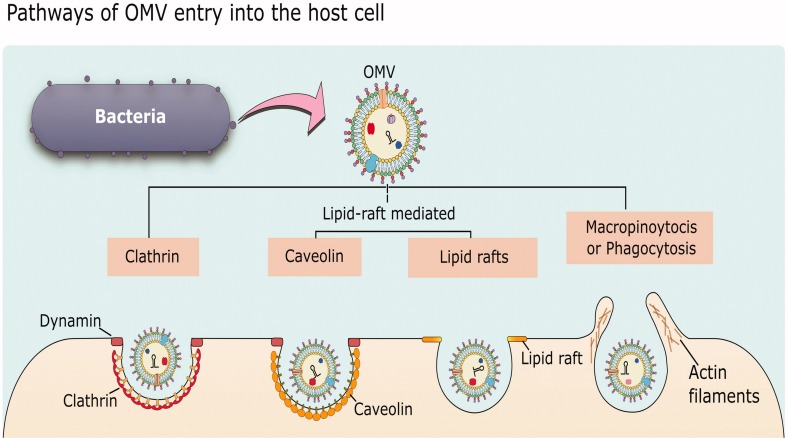
Mechanisms of OMV entry into host cells. OMVs can enter by clathrin-mediated endocytosis, lipid-raft-mediated endocytosis that may or may not be dependent on caveolin, or by macropinocytosis or phagocytosis which are more general pathways for uptake of material from the environment. Reproduced with permission from Anand and Chaudhuri (2016).

While there is abundant evidence that MVs can enter host cells and release their cargo to affect host cell physiology, the specific mechanisms underlying how MVs associate with and are taken up by host cells are still not fully understood. The following section will give a general overview of uptake pathways and then highlight some representative examples of MV entry into host cells to illustrate our current understanding of the processes involved and how some bacteria may exploit these processes to cause infection. In general, there are five different endocytic pathways by which MVs can be taken into non-phagocytic host cells: macropinocytosis, clathrin-mediated endocytosis, caveolin-mediated endocytosis, lipid raft-mediated endocytosis, and direct membrane fusion. Examples exist of MVs entering by each of these pathways (reviewed in Mulcahy et al., 2014; Anand and Chaudhuri, 2016; O’Donoghue and Krachler, 2016; Figure 5).

**FIGURE 6:**
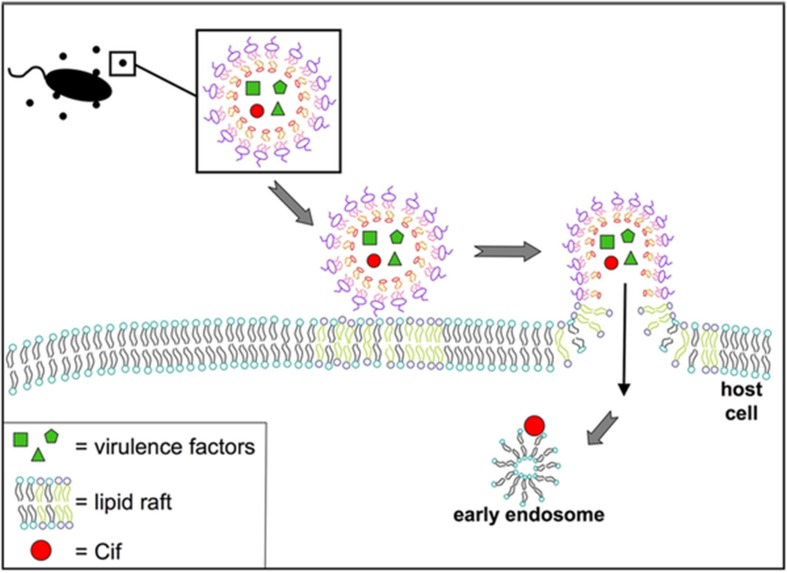
Model for fusion of *P. aeruginosa* OMVs with airway epithelial cells. Virulence factors and Cif are packaged into OMVs released by *P. aeruginosa.* These OMVs fuse with host cells at lipid raft microdomains in the plasma membrane. Reproduced with permission from Bomberger et al. (2009).

Actin-dependent macropinocytosis is driven by the polymerization of an actin ring below the cell membrane, resulting in a circular ruffled protrusion that eventually closes at the top to envelop a portion of the extracellular space (Bloomfield and Kay, 2016). It generally functions in cellular feeding and in antigen sampling by immune cells, but can also be exploited by viruses such as HIV and Ebola, or bacteria including *Salmonella* and *Listeria*, to enter cells (Maréchal et al., 2001; Saeed et al., 2010; Rosales-Reyes et al., 2012; Czuczman et al., 2014). This mechanism produces the largest endocytic vesicle of the various pathways (> 1 μm) (Amano et al., 2010). There is some evidence supporting OMV uptake through this mechanism, as uptake of *Pseudomonas* OMVs was reduced when cells were treated with cytochalasin D or wiskostatin, which inhibit actin polymerization (Figure 6; Bomberger et al., 2009). An actin-dependent pinocytic process is also involved in the uptake of *P. gingivalis* OMVs (Furuta et al., 2009).

Clathrin-mediated endocytosis can be triggered by a ligand binding to a cell surface receptor, and is followed by the formation of clathrin-coated pits which mature into vesicles (McMahon and Boucrot, 2011). Uptake via clathrin-mediated endocytosis can be tested for using chlorpromazine, which prevents formation of clathrin-coated pits, or with inhibitors of dynamin (such as dynasore), which is required for scission of the vesicle. Using these inhibitors, this pathway was shown to be a route of entry for a number of free virulence effectors including shiga toxin and cholera toxin, and for OMVs of several pathogens including *H. pylori*, *Brucella abortus*, *A. actinomycetemcomitans*, and multiple strains of *E. coli* (Sandvig and van Deurs, 2002; Pollak et al., 2012; Bielaszewska et al., 2013; Thay et al., 2014; Kunsmann et al., 2015; Vanaja et al., 2016).

Caveolin-mediated endocytosis involves membrane lipid raft domains that become enriched in caveolin protein, cholesterol, and sphingolipids, resulting in the formation of membrane invaginations called caveolae which are then internalized in a dynamin-dependent manner (Mulcahy et al., 2014). Endocytosis via the caveolin-mediated pathway is generally tested for using chemical inhibitors such as filipin or methyl−β cyclodextrin, which remove or disrupt cholesterol-rich membrane domains, or dynasore, which inhibits dynamin function to prevent internalization of vesicles. A number of bacterial species have been shown to use caveolin-mediated endocytosis as an alternate pathway to enter cells, including *E. coli*, *C. jejuni*, *S. typhimurium*, and *P. aeruginosa*, as well as a number of viruses (Machado et al., 2012). It has been suggested that this mechanism might be preferred by pathogens, as in contrast to clathrin-coated pits, bacteria internalized via caveolae are thought to avoid trafficking to lysosomes and subsequent degradation (O’Donoghue and Krachler, 2016). For example, *E. coli*, *Chlamydia trachomatis*, and *Leishmania chagasi* are able to avoid detection and persist within host cells when they enter through this pathway (Baorto et al., 1997; Norkin et al., 2001; Rodríguez et al., 2006). Entry by caveolin-mediated endocytosis has been shown for OMVs of some bacterial species, including *H. influenzae, Moraxella catarrhalis, E. coli*, and *V. cholerae* (Kesty et al., 2004; Chatterjee and Chaudhuri, 2011; Schaar et al., 2011; Sharpe et al., 2011). The specific interactions between bacterial ligands and host cell receptors that drive this internalization process have been identified in some cases; for example, cholera toxin produced by *V. cholerae* binds to the glycosphingolipid GM1 that is present in caveolin-enriched lipid rafts (Chatterjee and Chaudhuri, 2011). Similarly, enterotoxigenic *E. coli* vesicles containing the heat-labile enterotoxin LT1 also bind GM1 and are internalized via endocytic vesicles enriched in caveolin (Kesty et al., 2004). *M. catarrhalis* OMVs require interaction with the TLR2 receptor found within lipid rafts, though the specific bacterial ligand is not known (Schaar et al., 2011).

A fourth category of endocytic mechanism includes ones that do not depend on clathrin or caveolin but still utilize lipid rafts, which are membrane domains enriched in cholesterol and sphingolipids that are highly ordered and more rigid than the surrounding bilayer, and can accumulate signaling molecules (Mulcahy et al., 2014). Uptake via lipid rafts can be tested using filipin or methyl−β cyclodextrin to deplete cholesterol-rich domains in the membrane, though additional experiments are needed to distinguish between this mechanism and caveolin-mediated endocytosis, which also requires lipid rafts. OMVs from *P. aeruginosa, P. gingivalis, Vibrio vulnificus, A. baumannii, C. jejuni, V. cholerae* have all been shown to require lipid rafts for endocytosis using treatments with these inhibitors (Bauman and Kuehn, 2009; Furuta et al., 2009; Kim et al., 2010; Jin et al., 2011; Elmi et al., 2012; Mondal et al., 2016). Finally, OMVs are able to enter host cells through direct fusion with the host plasma membrane. This has been demonstrated for *P. aeruginosa, A. actinomycetemcomitans*, and *L. monocytogenes* using membrane-binding fluorescent dyes such as Rhodamine R-18, and appears to preferentially occur at lipid raft domains (Bomberger et al., 2009; Rompikuntal et al., 2012; Jäger et al., 2015).

It must be remembered that most of the chemical inhibitors of endocytic pathways have effects on more than one mechanism, thus it is often difficult to conclusively determine the pathway of uptake. Additionally, the mechanism of uptake of MVs for a given species may vary based on size and content – isolated MVs are heterogeneous in size and different sizes may enter more easily by different mechanisms, or particular lipid or protein cargo of the MVs might direct them to specific uptake pathways, as discussed in the following examples. The route of uptake has consequences for the delivery and eventual fate of the vesicle and its cargo, as will be discussed in the next section.

Some of the most extensive work to characterize uptake mechanisms for a given bacterial species has been done in *H. pylori*. Kaparakis et al. (2010) determined that *H. pylori* OMVs enter by a lipid raft-dependent mechanism, as disruption of lipid rafts with pharmacological inhibitors prevents OMV entry and OMV-triggered innate immune response in host cells. However, another study determined that entry of *H. pylori* OMVs is not dependent lipid rafts, but occurs via the clathrin-mediated pathway (Parker et al., 2010). Vesicles from a strain unable to produce the vacuolating cytotoxin VacA were more strongly affected by pharmacological inhibition of clathrin-dependent endocytosis, while OMVs containing VacA were shown to associate more strongly with host cells (potentially due to binding of VacA with cell surface components in lipid raft domains) and could still be internalized, though via an undetermined mechanism (Parker et al., 2010). Further work in this area by Olofsson et al. (2014) confirmed that uptake of *H. pylori* OMVs of a single strain occurs via both clathrin-dependent and clathrin-independent pathways. Membrane fluidity also appears to play a role in internalization, as uptake was reduced when membrane cholesterol was depleted, though not when it was sequestered to prevent formation of lipid rafts (Olofsson et al., 2014). Recently, it was determined that size is a determinant of uptake pathway for *H. pylori* OMVs: using careful controls, multiple pharmacological inhibitors, and siRNA knockdowns of various endocytic pathways, Turner et al. (2018) determined that a heterogeneously sized population of *H. pylori* could be internalized by macropinocytosis, clathrin-dependent, and caveolin-dependent mechanisms. Smaller OMVs (20 to 100 nm in size) were preferentially taken in by caveolin-dependent endocytosis, while larger OMVs (90 to 450 nm) were more dependent on clathrin- and dynamin-dependent processes (Turner et al., 2018). Importantly, this study also identified a role for vesicle size in determining protein content; smaller OMVs contained fewer proteins than large OMVs (28 vs. 137, as determined by LC-MS/MS). Vesicles of both sizes contained proteins associated with virulence and survival, but large OMVs also contained proteins involved in adhesion that were not found in small OMVs (Turner et al., 2018). This difference in protein content likely contributes to the variation in endocytic pathway favored by the differently sized OMVs. It was also independently shown by another group that bacterial growth stage determines OMV size and protein content for *H. pylori* (Zavan et al., 2019). This work highlights the complexity and variability of OMV biogenesis and interaction with host cells, and this represents an exciting area where further research is needed to better understand OMV biogenesis in the context of disease development.

**FIGURE 7:**
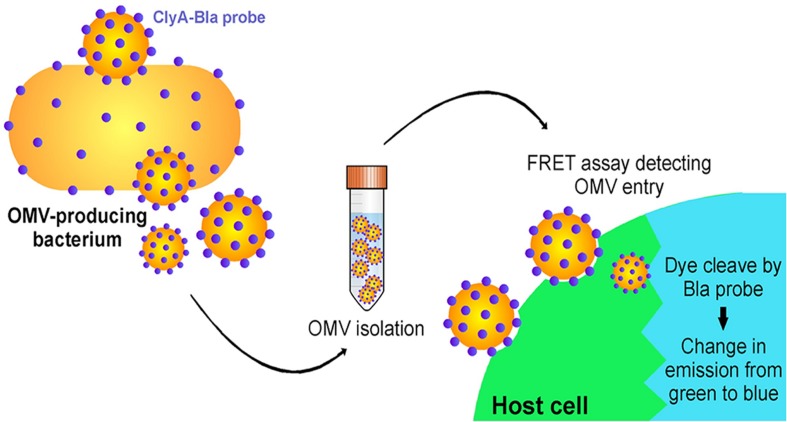
A FRET-based assay to monitor OMV entry. Target mammalian cells are loaded with a FRET-based reporter (indicated by the green color) that can be cleaved by an enzyme loaded within the OMV (Bla). Enzyme is released into the cytoplasm as OMVs fuse to the membrane of target cells, cleaving a ligand within the FRET reporter and leading to a shift in the emission wavelength (indicated by the blue color). Reproduced with permission from O’Donoghue et al. (2017).

**FIGURE 8:**
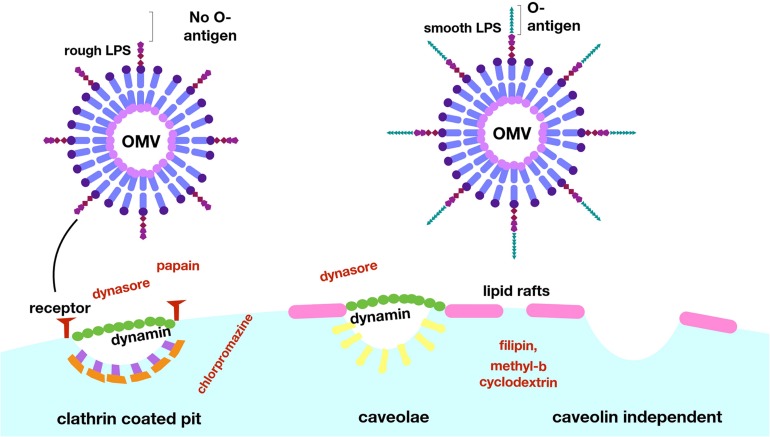
Lipopolysaccharide composition determines the mechanism of entry for OMVs into host cells. *E. coli* OMVs with LPS lacking the O-antigen enter via clathrin-coated pits, and uptake can be blocked experimentally with the use of dynasore or papain. OMVs with LPS containing the O-antigen enter via lipid rafts, a more efficient pathway, as evidenced by blocking of this uptake by filipin and methyl–β cyclodextrin. Reproduced with permission from O’Donoghue et al. (2017).

Another interesting example of OMV content affecting the pathway of uptake to host cells occurs in *E. coli* (O’Donoghue et al., 2017). O’Donoghue et al. (2017) developed a novel, highly sensitive probe that allows for real-time detection of OMV entry into mammalian cells via an OMV-bound probe protein that cleaves a FRET reporter to result in a change in fluorescence once it enters host cells (Figure 7). They used this probe to test the entry kinetics of OMVs from three different *E. coli* strains, two of which are pathogenic [enterohemorrhagic (ETEC), and enteroaggregative (EAEC)] and one laboratory strain. OMVs from pathogenic *E. coli* entered host cells faster than those from the non-pathogenic strain, and this difference was dependent on the O antigen, which is a variant of the outermost structural region of LPS that is found in numerous pathogenic strains of *E. coli*. OMVs that contained the O antigen had a higher rate of uptake over a longer period of time, thus they entered host cells more efficiently than OMVs from control strains lacking the antigen (O’Donoghue et al., 2017). Using pharmacological inhibitors of endocytic pathways, the authors determined that OMVs lacking the O antigen primarily enter through clathrin-mediated endocytosis, while those containing the O antigen enter by the faster lipid raft mechanism (Figure 8). In addition to increasing the uptake efficiency of OMVs, it is also possible that directing uptake to the lipid raft pathway has consequences for the eventual fate of the OMV and its cargo in the host cell, however, more research is needed to determine whether this is the case. This work provides important insight into the sort of specific adaptations that might be used by pathogenic bacteria to effectively colonize the host and represents a promising topic for further research to better understand these processes.

In addition to OMV content, the mechanism of OMV biogenesis can also have an effect on how they are internalized by mammalian cells. The probiotic strain *E.* coli Nissle 1917 (EcN) produces OMVs that enter host cells via clathrin-mediated endocytosis. In laboratory contexts, hypervesiculating bacterial mutants are often utilized to achieve greater OMV production and isolation, including the *tolR* mutant of EcN. This mutant produces greater than 30-fold more OMVs and the protein profile of the OMVs is generally similar to wild type, however, TEM observation indicated that they are much more structurally heterogeneous and have membrane morphologies not found in wild type OMVs (Pérez-Cruz et al., 2016). As compared to OMVs from the wild type strain, OMVs from the *tolR* mutant still enter via clathrin-mediated endocytosis, but far fewer are actually internalized as they have reduced ability to bind epithelial cell membranes (Pérez-Cruz et al., 2016). The authors suggest that perhaps only “conventionally produced” OMVs may be able to efficiently interact with host cells. Differences in OMV protein content, immunogenicity, and efficiency of uptake to host cells have also been reported for Δ*tolB* and Δ*pal* mutants of *H. pylori*, further highlighting the importance of OMV structure and cargo content in driving their role in their function (Turner et al., 2015).

**FIGURE 9:**
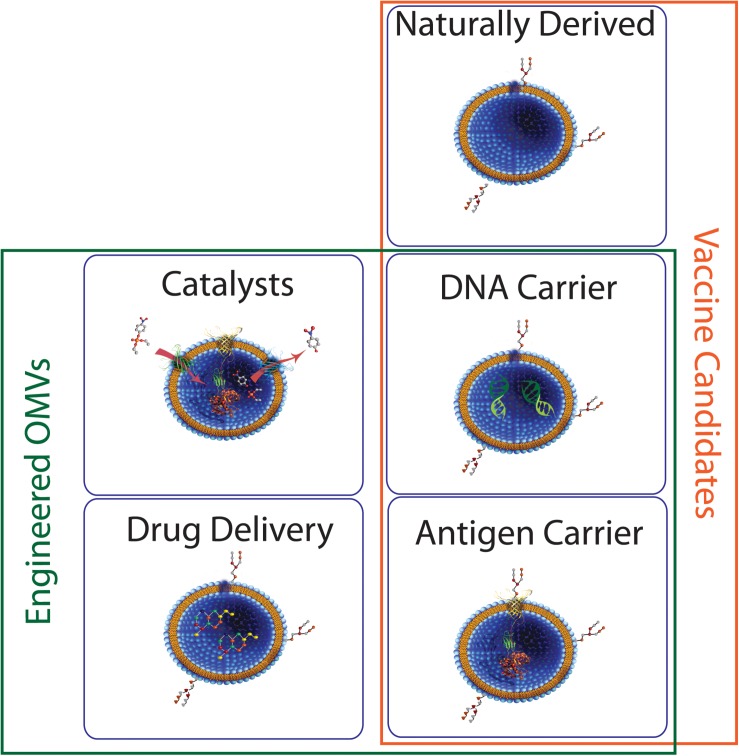
Potential roles for bacterial MVs. Membrane vesicles isolated directly from bacterial culture have seen some success as vaccine candidates. Engineered MVs have also been used for successful expression and/or delivery of biomolecules such as proteins and nucleic acids, indicating potential in areas such as vaccine development and industrial catalysis.

Taken together, the above examples highlight the importance not just of determining which endocytic pathway(s) are responsible for uptake of MVs from a given bacterial species in a single context, but of more exhaustive studies that take into account variation in bacterial growth rate, environment, genotype, and mechanism of biogenesis if we are to gain a fuller understanding of their interaction with host cells. OMVs have the potential for use in numerous biotechnological applications including vaccines and drug delivery (Figure 9) and it is likely that such uses would benefit from strain modifications that increase production and uptake, thus it will be critical to gain a greater understanding of how alterations in OMV biogenesis might affect the eventual function of the vesicles and their ability to interact with host cells. Further research in this area will greatly expand our ability to identify MV function in pathogenesis and to exploit the properties of MVs for biotechnological uses.

### Fate and Function of MV Cargo Inside Host Cells

While we now have detailed information on the various processes that govern uptake of MVs into host cells, a gap remains in our understanding of what happens to MVs and their cargo after internalization. Once inside host cells, MVs must escape degradation long enough to release their cargo and that cargo must itself escape degradation for long enough to perform its function. In general, MVs that are taken up in endocytic vesicles progress through the endolysosomal pathway and are eventually broken down in lysosomes, though vesicles internalized through caveolin-mediated endocytosis are instead delivered to the endoplasmic reticulum/Golgi complex (Pelkmans and Helenius, 2002; O’Donoghue and Krachler, 2016). As mentioned above, it has been suggested that this is the favored mechanism of uptake for bacterial MVs for this reason, as it may allow MVs and their cargo to escape degradation (O’Donoghue and Krachler, 2016). The specific mechanisms that govern MV persistence inside host cells and how their associated virulence factors (and other components, in the case of beneficial interactions) are released and carry out their functions remain unclear, though a few recent studies have provided insight in this area.

**FIGURE 10:**
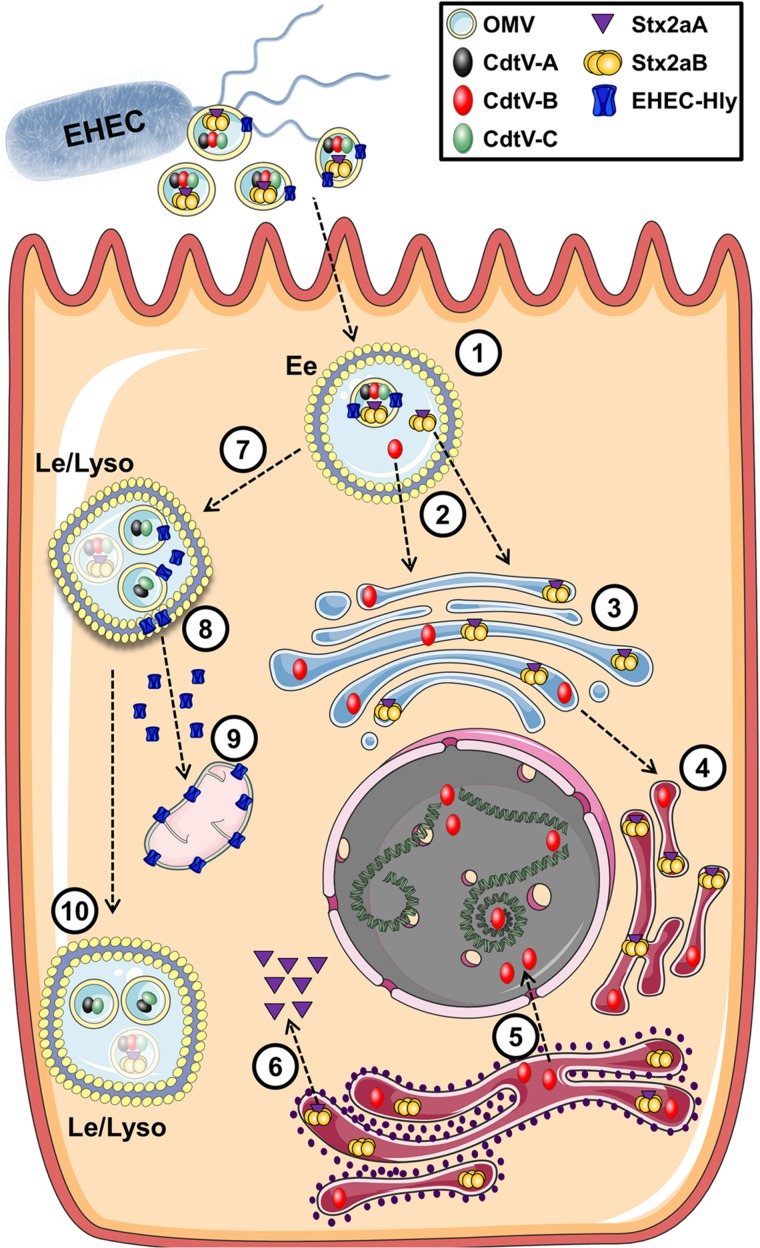
Intracellular trafficking of bacterial toxins delivered via OMVs. In this example toxins contained in OMVs of *E. coli* O157 are endocytosed into an endosome (1) where the cargo toxins separate into individual subunits (2) which escape the endosome and migrate to the Golgi (3) and endoplasmic reticulum (4). These compounds eventually make their way to their cellular targets of DNA (5) or the ribosome (6). Additional cytotoxic components within the OMV enter the late endosome (7) and eventually escape to interact with the mitochondria (8,9) while remaining OMV-associated components are degraded in the lysosome (10). Reproduced with permission from Bielaszewska et al. (2017).

Enterohemorrhagic *E. coli* (EHEC) O157 strains are the leading serogroup of *E. coli* that cause human disease, namely hemolytic-uremic syndrome. They have been extensively studied and a number of virulence factors identified in OMVs that contribute to their ability to cause disease, including shiga toxin (Stx2A), cytolethal distending toxin V (CdtV), and hemolysin (ETEC-Hly) (Karch et al., 2005; Kunsmann et al., 2015). Two excellent and detailed studies by Bielaszewska et al. (2013, 2017) used a combination of proteomics, microscopy, immunoblotting, and bioassays to produce a comprehensive analysis of the fate of EHEC OMVs and their associated virulence factors after internalization. EHEC OMVs are internalized via dynamin-dependent endocytosis and in part by clathrin-mediated endocytosis, after which they follow the endocytic pathway from early endosomes to lysosomes, where they are degraded. Their toxin cargo separates from the vesicles during intercellular trafficking. The shiga toxin Stx2a separates from the OMVs in early endosomes, possibly due to the slightly lowered pH in those compartments. It then associates with its receptor globotriaosyl ceramide within lipid raft domains of endocytosed membranes, and is trafficked to the endoplasmic reticulum and Golgi and then to the cytosol. CdtV consists of three subunits which have different fates: CdtV-B, which is a DNase-like subunit, also separates from the OMVs in early endosomes and travels to the endoplasmic reticulum and then to the nucleus, where it causes DNA damage that leads to G2 cell cycle arrest. In contrast, the CdtV-A and CdtV-C subunits remain with the OMVs and are sorted to lysosomes for degradation. Cellular lethality results from G2 cell cycle arrest due to CdtV-B subunit activity, followed by caspase-9-activated apoptosis triggered by Stx2A (Figure 10; Bielaszewska et al., 2017). EHEC-Hly travels with the OMVs to lysosomes, where the acidic pH allows it to escape into the cytosol and subsequently to the mitochondria where it triggers apoptotic cell death (Bielaszewska et al., 2013). Interestingly, ETEC-Hly is also secreted in free form, in which it causes lysis of microvascular endothelial cells, presumably by inserting itself into the host cell plasma membrane and causing formation of pores that lead to lysis (Aldick et al., 2007). However, OMV-associated hemolysin is more stable and has prolonged activity, indicating that it is the most efficient form of the toxin and highlighting the importance of understanding the mechanisms of OMV-based virulence factor delivery (Aldick et al., 2009).

### Beneficial Interactions of MVs With Host Cells

While the majority of research on MVs and their interaction with host cells has been done on pathogenic species, there are also a number of relatively recent reports that detail the effects of MVs from commensal bacteria on host cells, particularly those that reside in the digestive tract. Probiotic bacteria have beneficial effects on gut function including prevention or reduction in symptoms of certain diseases, often through modulation of immune function of the host but also through competitive exclusion of pathogenic bacteria and strengthening of the epithelial cell barrier in the gut (Ohland and MacNaughton, 2010; Plaza-Díaz et al., 2017). The exact mechanisms by which they exert these effects are still under study, but there is increasing awareness that communication between probiotic bacteria and host cells is mediated by MVs. Direct physical contact between bacteria in the gut and the intestinal epithelium is inhibited by a thick layer of mucus, thus for a long time it was unclear how probiotic bacteria are able to exert their effects on the host. It is becoming increasingly clear that MVs, which are able to pass through the mucus layer, provide at least one important avenue of communication between microbes and their hosts. In this section we will provide representative examples of beneficial effects of MVs from commensal bacteria on host cells. This topic has been recently reviewed by others as well, and their work may be referred to for additional information (Cecil et al., 2019; Molina-Tijeras et al., 2019).

The first report of beneficial effects of MVs from a commensal bacterium came in 2012, when Shen et al. (2012) showed that OMVs from *Bacteroides fragilis* could induce immunomodulatory effects and prevent development of experimentally induced colitis in mice. Species of the genus *Bacteroidetes* are the most common commensal organisms in the human digestive tract, and *B. fragilis* had been previously shown to protect against inflammatory bowel disease and multiple sclerosis in animal models (Mazmanian et al., 2008; Ochoa-Repáraz et al., 2010). This protective effect was due to a single molecule, Polysaccharide A (PSA), which both induces the development of regulatory T cells that suppress inflammation and also suppresses immune responses directed against *B. fragilis* (Ochoa-Repáraz et al., 2010; Round and Mazmanian, 2010). As PSA is a large polysaccharide and *B. fragilis* is not thought to possess genes encoding secretion machinery for PSA, it had been a mystery as to how it is delivered to host cells (Cerdeño-Tárraga et al., 2005). Shen et al. (2012) provided evidence that it is packaged into OMVs, and orally administered OMVs containing PSA provided protection against experimentally induced colitis in mice. The OMVs were internalized by bone marrow-derived dendritic cells (DC) in a toll-like receptor 2-dependent manner and could induce expression of IL-10, a protective anti-inflammatory molecule. Dendritic cells specialize in antigen capture and presentation to T-cells, and as such those DCs that internalize *B. fragilis* OMVs induce production of regulatory T-cells which also make IL-10 and provide the protective effect against colitis (Shen et al., 2012). OMVs of *B. fragilis* also cause a reduction in pro- inflammatory (IFNγ) cytokines while increasing expression of anti-inflammatory (IL-4 and IL-10) cytokines in human intestinal epithelial (Caco-2) cells (Ahmadi Badi et al., 2019). An interesting question that remains to be investigated is the mechanism that accounts for PSA from *B. fragilis* found in the gut providing protection against multiple sclerosis (Ochoa-Repáraz et al., 2010). How does this molecule produced in the gut have an effect on the central nervous system? As MVs from species of the intestinal microbiota are able to migrate into the bloodstream, it is tempting to speculate that PSA-containing OMVs of *B. fragilis* may interact with dendritic cells in organs outside the digestive tract (Park et al., 2017). In support of this hypothesis, it was recently demonstrated that injection of *L. plantarum* MVs into the bloodstream of mice could reduce stress-induced depressive behaviors and restore expression of BDNF, a neurotrophic factor that is reduced during depression, effects that had been previously demonstrated for oral supplementation with *Lactobacillus* species (Choi et al., 2019).

Outer membrane vesicles from the probiotic *E. coli* strain Nissle 1917 (EcN) have also been demonstrated to have a protective effect against colitis (Alvarez et al., 2016; Fábrega et al., 2017). Pretreatment of mice with OMVs prior to chemical induction of colitis results in reduced expression of the inflammatory enzymes cyclooxygenase (COX)-2 and inducible nitric oxide synthase (iNOS), reduced production of inflammatory cytokines, and an overall decrease in symptoms (Fábrega et al., 2017). It was also shown by Alvarez et al. (2016) that EcN OMVs strengthen the intestinal barrier and reduce permeability by causing upregulation of the tight-junction proteins ZO-1 and claudin-14, and down-regulation of claudin-2 (which induces channel formation, contributing to leakiness).

Similar immunomodulatory effects and strengthening of the intestinal barrier against colitis have been reported for OMVs of other Gram-negative commensal species, including *Bacteroides vulgaris* and the relatively recently identified probiotic *Akkermansia* (Kang et al., 2013; Chelakkot et al., 2018; Maerz et al., 2018). This is also the case for multiple Gram-positive species of the genus *Lactobacillus*, including *L. rhamnosus, L. sakei*, and the kefir-derived strains *L. kefir*, *L. kefiranofaciens*, and *L. kefirgranum* (Al-Nedawi et al., 2015; Seo et al., 2018; Yamasaki-Yashiki et al., 2019). These effects occur through various mechanisms, including stimulation of dendritic cells, modulation of inflammatory cytokine expression, reduction in oxidative stress, and stimulation of IgA production, which regulates the composition of the gut microbiome and contributes to strengthening of the epithelial cell barrier.

Beyond the above examples in which membrane vesicles have been clearly shown to mediate probiotic effects, similar stimulation of dendritic cells and induction of regulatory T-cells has been reported for a number of other probiotic species including *Lactobacillus reuteri, Lactobacillus casei*, *Bifidobacterium animalis* and *Bifidobacterium adolescentis.* While not yet confirmed experimentally, it is likely that these effects occur at least in part through MVs (Smits et al., 2005; Baba et al., 2008). In support of this hypothesis, p40 and p75, two proteins found in *L. casei* and *Lactobacillus rhamnosus* that have anti-apoptotic and cell protective effects on human intestinal epithelial cells, have been identified in MVs isolated from *L. casei* cultures (Bäuerl et al., 2010; Domínguez Rubio et al., 2017).

In addition to their protective effects against colitis, the anti-inflammatory properties of membrane vesicles can protect against allergic responses. MVs of the Gram-positive commensal *Bifidobacterium bifidum* have been shown to stimulate dendritic cells, resulting in production of regulatory T-cells and IL-10, and this has a protective effect against allergies (Kalliomäki and Isolauri, 2003; López et al., 2012). Similar alleviation of food allergy symptoms has been observed for *Bifidobacterium longum*, which induces apoptosis of mast cells (Kim et al., 2016). This has led to speculation that MVs of this or other probiotic species could be used as adjuvants for allergen-specific immunotherapy (López et al., 2012). This may be true for other conditions as well, including obesity; for example, OMVs of *Akkermansia muciniphila* were very recently shown to reduce weight gain, adipose tissue inflammation, gut barrier permeability, blood glucose, and blood cholesterol when fed to mice given a high fat diet (Ashrafian et al., 2019). Interestingly, some of these effects were stronger in response to the OMVs than in response to the parent bacteria.

There is evidence that probiotic bacteria may have cancer prevention properties, particularly against colon cancer (Commane et al., 2005; Uccello et al., 2012; dos Reis et al., 2017). This effect is due at least in part to pro-apoptotic factors released by the bacteria (reviewed in Uccello et al., 2012; Dasari et al., 2017). For example, *L. reuteri* promoted apoptosis and suppressed expression of cell proliferative and anti-apoptotic proteins in cells treated with tumor necrosis factor (TNF), a pro-inflammatory cytokine that may be involved in inflammation-induced carcinogenesis (Iyer et al., 2008). Pretreatment of rats with *L. rhamnosus* GG in combination with the NSAID drug celecoxib prior to chemically induced carcinogenesis reduced the expression of pro-carcinogenic markers and induced expression of pro-apoptotic proteins, and reduced the number of colonic tumors observed (Sharaf et al., 2018). Similarly, treatment of multiple human cancer cell lines with *Bacillus coagulans* resulted in decreased proliferation and increased expression of apoptotic markers (Madempudi and Kalle, 2017). Interestingly, this effect was observed for heat-killed culture supernatant as well as for live bacterial cells, so it is likely that some or all of these anti-cancer effects are mediated by MVs. In support of this, it was recently shown that purified MVs from *L. rhamnosus* have significant cytotoxic effects on hepatic cancer cells (Behzadi et al., 2017). As membrane vesicles derived from the microbiota in the gastrointestinal tract can travel to the liver and other nearby organs through the bloodstream, this provides further evidence for the potent anti-cancer potential of probiotic bacteria and their membrane vesicles (Park et al., 2017). As probiotic bacteria generally do not have deleterious effects on the host, use of these bacteria and/or their MVs may make possible the development of new cancer treatments that do not also cause the damaging side effects of traditional chemotherapeutic agents (Behzadi et al., 2017; Sharaf et al., 2018).

Probiotic bacteria also have protective effects against pathogen infection. The mechanisms underlying this protection are not fully understood, but occur through a number of mechanisms including (but not limited to) production of antimicrobial substances such as bacteriocins and other metabolites, competitive exclusion of pathogens, binding of pathogen-generated toxins, or through modulation of host immune signaling (Oelschlaeger, 2010). Again, there is mounting evidence that MVs play a role in this communication between probiotic bacteria and host cells. For example, *Lactobacillus acidophilus* has been demonstrated to activate immune signaling pathways against Gram-positive bacteria in *Caenorhabditis elegans* and prolongs survival after challenge with vancomycin-resistant *Enterococcus faecium* (VRE) (Kim and Mylonakis, 2012). It was later shown by the same group that MVs derived from *L. plantarum* induced upregulation of multiple defense genes in human Caco-2 cells, and when given to *C. elegans*, resulted in upregulation of homologous defense genes and protection against VRE (Li et al., 2017). Similarly, MVs isolated from *L. sakei* could stimulate production of IgA by Peyer’s patch cells in the mouse intestine (Yamasaki-Yashiki et al., 2019). Very recently, MVs of *Lactobacillus crispatus* and *Lactobacillus gasseri* were demonstrated to inhibit HIV-1 infection of human cervico-vaginal and tonsillar tissues *ex vivo* (Ñahui Palomino et al., 2019). This effect is mediated in part by a reduction in exposure of the Env protein that mediates virus-cell interactions in MV-treated viral particles, as well as by several other EV-associated bacterial proteins and metabolites whose specific functions in protection are yet to be identified (Ñahui Palomino et al., 2019). Other probiotic bacteria have been shown to have protective effects based on their modulation of host immune responses, including *E.* coli Nissle 1917 and multiple *Lactobacillus* and *Bifidobacterium* species, against pathogens including enteropathogenic *E. coli, S. typhimurium*, and *P. gingivalis*, and *C. albicans*, to name only a few examples (Zyrek et al., 2007; Castillo et al., 2011; Albuquerque-Souza et al., 2019), reviewed in Kosgey et al. (2019) and Sanders et al. (2019). It is likely that some or all of these effects occur at least in part through MVs, and further experimentation in that area may lead to the development of MV-based therapeutics.

## Section 3: Future Directions of Bacterial MVs

As has been described in preceding sections, naturally derived MVs and OMVs play an important role in microbial community regulation and interactions with host cells and tissues. With a greater understanding of the microbial systems that control packaging of MVs and the environmental conditions that initiate these genetic and cellular systems, researchers may one day be able to develop microbial “cocktails” that convey specific benefits or advantages to the individual as a form of personalized probiotic. One can easily envision the short-term benefits of these advancements such as post-surgical supplements that stimulate host immune responses for defense against opportunistic pathogens or long-term alleviation of conditions such as Irritable Bowel Syndrome (IBS) or other food allergies through introduction of organisms capable of colonizing the gut microbiome and continuously producing and releasing biomolecules that alleviate these conditions. In many instances, these successes may be realized using natural bacterial flora as seen by the diverse contingent of organisms already described in the literature. Alternatively, the potential for designer organisms tailored to produce specific components and release them on demand is also on the horizon. In these instances, implementing engineered MVs as delivery vehicles allows for the potential long-range delivery to specific cell types and tissues while affording protection from environmental conditions including pH, proteases, nucleases, and other biomolecules that could reduce the efficacy of the recombinant biomolecule (Figure 9).

### Engineering Bacterial Membrane Vesicles

In most microbial species, the cellular and genetic machinery that enable MV production and packaging are poorly understood and not typically defined, rather directed packaging is often described in phenomenological terms. Despite this lack of molecular definition of cellular systems for MV loading, many researchers have shown that localization of recombinant proteins to the periplasmic space is often sufficient to ensure MV encapsulation of the recombinant proteins. As an example, Kesty et al. simultaneously demonstrated MV loading of green fluorescent protein (GFP) and cellular uptake using a surface localized bacterial peptide (Kesty and Kuehn, 2004; Kesty et al., 2004). In this instance, simple over-expression of recombinant protein was sufficient to drive OMV encapsulation in *E. coli*. This mechanism, however, is not always successful. As described by Alves et al. (2015) in their works with an organophosphate hydrolyzing enzyme, successful encapsulation of the enzyme could only be achieved through a more active loading via a membrane anchor and protein-protein interaction. The researchers were unable to determine why this particular enzyme was unable to passively load into nascent OMVs, however, this phenomenon has been described for both native and recombinant proteins by others. Comparison of the proteome of parental cells and their MVs from multiple bacterial species has shown that in many cases the abundance of proteins in the periplasm and outer membrane do not correlate to proportional concentrations within the MVs (Lee et al., 2007, 2008; Dean et al., 2019).

**FIGURE 11:**
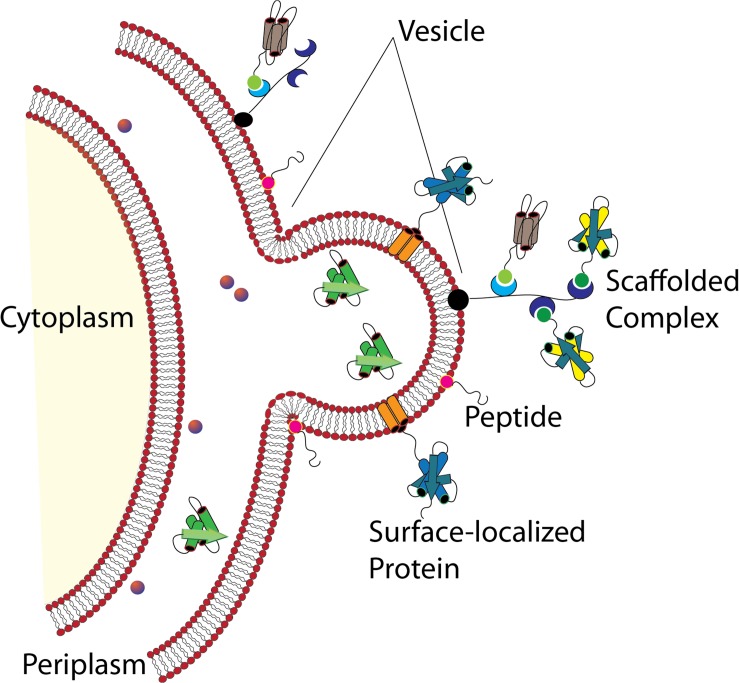
Modification of OMVs. A number of strategies have been employed to functionalize bacterial MVs both at the surface and within the lumen. MVs can be passively loaded through periplasmic localization of recombinant proteins (green) or through other anchoring mechanisms that can position recombinant proteins both internally or externally (blue). Peptides and proteins have been anchored to the surface for targeting and purification (pink sphere) while protein scaffolds have been used to assemble enzyme cascades.

To realize MVs and OMVs as delivery vehicles researchers have had to develop strategies to ensure successful encapsulation of recombinant proteins and biomolecules using an active form of MV loading (Figure 11). Some success has been achieved for active loading via fusions to abundant OMV proteins such as the ClyA toxin protein of *E. coli* (Wai et al., 2003; Chen et al., 2010; Baker et al., 2014) or through recombinant membrane spanning proteins that provide a mechanism for decorating the exterior of OMVs with recombinant proteins of interest (Gujrati et al., 2014; Park et al., 2014; Alves et al., 2017; Su et al., 2017). Systems can also be designed *de novo* allowing researchers to develop modular systems that allow for rapid exchange of cargo proteins as seen in several publications by Alves et al. (2015, 2016, 2018).

The use of engineered bacteria for therapeutic purposes may still be quite a few years off, however, these foundational studies in MV engineering will undoubtedly aid in moving the community forward. Membrane vesicles from several bacterial species have already seen success as potential vaccine candidates as summarized in several excellent reviews (Cai et al., 2018; Tan et al., 2018; Caruana and Walper, 2020). Additionally, engineered OMVs displaying recombinant antigens designed to serve as both adjuvant and antigen are emerging as another vaccine concept as shown in current efforts by several groups and reviewed by van der Pol et al. (van der Pol et al., 2015; Chen et al., 2016; Irene et al., 2018; Stevenson et al., 2018). Many of the other potential therapeutic applications such as community regulation and immunoregulation have been discussed in the preceding sections and are reviewed elsewhere by Baker et al. (2014) and Qing et al. (2019).

The fields of synthetic biology and metabolic engineering will continue to enable microbial engineering for applications ranging from biomanufacturing to improving human health and performance. Whether in traditional laboratory strains or within new microbial chassis species, the role of MVs in enabling these processes will be invaluable as researchers are able to customize non-replicating biological particles that can be field-deployed without concerns for releasing genetically modified organism, catalysts that can be used for Green manufacturing practices, or even tailoring vaccines and medicines that no longer require a consistent cold-chain for delivery to austere environments. Advances such as these have already been described in the literature and will serve as foundations for what is yet to come.

## Author Contributions

Both authors listed have made a substantial, direct and intellectual contribution to the work, and approved it for publication.

## Conflict of Interest

The authors declare that the research was conducted in the absence of any commercial or financial relationships that could be construed as a potential conflict of interest.
